# Correction: Longitudinal mediation analysis of the factors associated with trajectories of posttraumatic stress disorder symptoms among postpartum women in Northwest Ethiopia: Application of the Karlson-Holm-Breen (KHB) method

**DOI:** 10.1371/journal.pone.0320696

**Published:** 2025-03-20

**Authors:** The PLOS One Staff

## Notice of republication

This article was republished on February 24, 2025, to correct errors in Table 5 that were introduced during the typesetting process. The publisher apologizes for the errors. Please download this article again to view the correct version. The originally published, uncorrected article and the republished, corrected articles are provided here for reference.

## Supporting information

S1 File Originally published, uncorrected article. (PDF)

S2 File Republished, corrected article. (PDF)
