# Supplementary material for: Longitudinal mediation analysis of the factors associated with trajectories of posttraumatic stress disorder symptoms among postpartum women in Northwest Ethiopia: Application of the Karlson-Holm-Breen (KHB) method
Source: PLoS One. 2022 Apr 11;17(4):e0266399. doi: 10.1371/journal.pone.0266399 (PMC9000968; doi:10.1371/journal.pone.0266399)
Supplement: Attachment — Submitted filename: Response to Reviewers.docx [file pone.0266399.s002.docx]

**Author’s response for reviewers’ comments**

**Response for Editor(s)' Comments:**

**Comments:**

Journal Requirements:

1. When submitting your revision, we need you to address these additional requirements.

Please ensure that your manuscript meets PLOS ONE's style requirements, including those for file naming. The PLOS ONE style templates can be found at 
<https://journals.plos.org/plosone/s/file?id=wjVg/PLOSOne_formatting_sample_main_body.pdf> and 
<https://journals.plos.org/plosone/s/file?id=ba62/PLOSOne_formatting_sample_title_authors_affiliations.pdf>

**Response:**

The manuscript is formatted in line with the journal requirement.

Now the manuscript meets PLOS ONE's style requirements, including those for file naming (see the clean copy and marked copy of the revised document)

**Comments:**

2. Please note that in order to use the direct billing option the corresponding author must be affiliated with the chosen institute. Please either amend your manuscript to change the affiliation or corresponding author, or email us at [plosone@plos.org](mailto:plosone@plos.org) with a request to remove this option.

**Response:** Please remove the direct billing option. I have sent an email to [plosone@plos.org](mailto:plosone@plos.org) with a request to remove this option. I was not aware of it.

**Comments:**

3. In your Data Availability statement, you have not specified where the minimal data set underlying the results described in your manuscript can be found. PLOS defines a study's minimal data set as the underlying data used to reach the conclusions drawn in the manuscript and any additional data required to replicate the reported study findings in their entirety. All PLOS journals require that the minimal data set be made fully available. For more information about our data policy, please see <http://journals.plos.org/plosone/s/data-availability>.

Upon re-submitting your revised manuscript, please upload your study’s minimal underlying data set as either Supporting Information files or to a stable, public repository and include the relevant URLs, DOIs, or accession numbers within your revised cover letter. For a list of acceptable repositories, please see <http://journals.plos.org/plosone/s/data-availability#loc-recommended-repositories>. Any potentially identifying patient information must be fully anonymized.

Important: If there are ethical or legal restrictions to sharing your data publicly, please explain these restrictions in detail. Please see our guidelines for more information on what we consider unacceptable restrictions to publicly sharing data: <http://journals.plos.org/plosone/s/data-availability#loc-unacceptable-data-access-restrictions>. Note that it is not acceptable for the authors to be the sole named individuals responsible for ensuring data access.

We will update your Data Availability statement to reflect the information you provide in your cover letter.

**Response:**

Now, I have uploaded my study’s minimal underlying data set as Supporting Information files.

**Comments:**

4.We note that you have stated that you will provide repository information for your data at acceptance. Should your manuscript be accepted for publication, we will hold it until you provide the relevant accession numbers or DOIs necessary to access your data. If you wish to make changes to your Data Availability statement, please describe these changes in your cover letter and we will update your Data Availability statement to reflect the information you provide.

**Response:**

Now, I have already uploaded the minimal dataset as supporting information. Previously I understand the word “repository information for my data at acceptance” as to mean that uploading the supporting information of data file up on acceptance. So, I didn’t understand it clearly. So, please update my Data Availability statement in line with providing minimal dataset as supporting information.

**Comments:**

5. Please review your reference list to ensure that it is complete and correct. If you have cited papers that have been retracted, please include the rationale for doing so in the manuscript text, or remove these references and replace them with relevant current references. Any changes to the reference list should be mentioned in the rebuttal letter that accompanies your revised manuscript. If you need to cite a retracted article, indicate the article’s retracted status in the References list and also include a citation and full reference for the retraction notice.

**Response:**

Now, I have updated my references list in line with the journal’s requirement. No retracted papers have been used

**Comments:**

6. We noticed you have some minor occurrence of overlapping text with the following previous publication(s), which needs to be addressed:

- <https://openaccess.city.ac.uk/id/eprint/19244/1/Running%20head%20Trajectories%20of%20birth-related%20PTSD.pdf>
- <https://journals.sagepub.com/doi/10.1177/1359105318787018>

The text that needs to be addressed involves the Introduction.

In your revision ensure you cite all your sources (including your own works), and quote or rephrase any duplicated text outside the methods section. Further consideration is dependent on these concerns being addressed.

**Response:**

Now, some of the overlapping texts in few paragraphs are rephrased and all sources have been cited accordingly in the introduction section.

**Comments:**

Additional Editor Comments:

Dear authors -
The reviewers were enthusiastic about your manuscript. However, there were some modifications that were suggested to improve the manuscript. Please see their comments and revise your manuscript appropriately.

**Response:**

See my response for reviewers’ comment bellow.

**Response for reviewers’ comments**

**Comments:**

Reviewers' comments:

Reviewer's Responses to Questions

**Comments to the Author**

1. Is the manuscript technically sound, and do the data support the conclusions?

The manuscript must describe a technically sound piece of scientific research with data that supports the conclusions. Experiments must have been conducted rigorously, with appropriate controls, replication, and sample sizes. The conclusions must be drawn appropriately based on the data presented.

Reviewer #1: Yes

Reviewer #2: Yes

**Response:** It is ok

2. Has the statistical analysis been performed appropriately and rigorously?

Reviewer #1: Yes

Reviewer #2: I Don't Know

**Response:** It is shown in the methods and results section.

3. Have the authors made all data underlying the findings in their manuscript fully available?

The [PLOS Data policy](http://www.plosone.org/static/policies.action#sharing) requires authors to make all data underlying the findings described in their manuscript fully available without restriction, with rare exception (please refer to the Data Availability Statement in the manuscript PDF file). The data should be provided as part of the manuscript or its supporting information, or deposited to a public repository. For example, in addition to summary statistics, the data points behind means, medians and variance measures should be available. If there are restrictions on publicly sharing data—e.g. participant privacy or use of data from a third party—those must be specified.

Reviewer #1: Yes

Reviewer #2: No

**Response:** Now, I have uploaded the minimal dataset.

4. Is the manuscript presented in an intelligible fashion and written in standard English?

PLOS ONE does not copyedit accepted manuscripts, so the language in submitted articles must be clear, correct, and unambiguous. Any typographical or grammatical errors should be corrected at revision, so please note any specific errors here.

Reviewer #1: Yes

Reviewer #2: Yes

**Response:** It is ok.

5. Review Comments to the Author

Please use the space provided to explain your answers to the questions above. You may also include additional comments for the author, including concerns about dual publication, research ethics, or publication ethics. (Please upload your review as an attachment if it exceeds 20,000 characters)

**Response for 1^st^ reviewer comments**

**Comments:**

Reviewer #1: Thank you for the opportunity to review this manuscript! I find the results obtained extremely valuable and important. The procedure and the results are clearly described. Women with still birth were included in the sample. I suggest to mention this group in the results section, as antenatal loss is known to be a strong predictor of PTSD. And I have a question about the age variable. The inclusion criterion was 15 y.o. Were there any differences in the teenage mothers group regarding pospartum PTSD risks?

**Response**

Now, I have included the result for teenage mothers’ group in Table 1 and still birth in Table 3. But, since the number of events were rare, there was no significant association with the PTSD trajectory group. Please, see the number of events(frequencies) for these variables in the specified tables, to appreciate how rare were these variables.

**Response for 1^st^ reviewer comments**

**Comments:**

Reviewer #2: The authors investigated the trajectory of PTSD symptoms in Ethiopian women postpartum. They also conducted a mediation analysis to determine the factors associated with each trajectory. They found 4 trajectories: 1) no symptoms, 2) subthreshold, 3) transient symptoms, and 4) persistent symptoms.

Various factors were associated with the three trajectories comprising PTSD symptoms compared to symptoms, including high levels of depression and anxiety.

The manuscript is clear and comprehensive but very lengthy at times. The methods, results and discussion sections could be shortened. Some details can be moved to Online Supplements. Otherwise, the research question is of relevance to the field. While I cannot comment on the statistical methods, over all the approach seems adequate, the results are informative and the conclusion are primarily reasonable.

**Response:**

The improve the length of the manuscript, the following portions were removed from the specified sections of the manuscript (see below):

**Abstract section (result subsection):** The following paragraphs (sentences) were removed.

Prevalence rates of PTSD symptoms were 9.7%, 6.8% and 3.5% at the 6^th^, 12^th^ and 18^th^ week of postpartum respectively.

**Method section (Data processing and analysis subsection):** The following paragraphs (sentences) were removed.

Statistically, group-based trajectory models use maximum likelihood estimation to estimate both the trajectory of each group (modeled as a function of time using flexible polynomials) and the expected population-level distribution of each group that creates the best fit for the observed data (64, 65). Since the number of groups and the order of the trajectory polynomials (i.e., linear, quadratic, cubic) are not actually known a priori (but must be prespecified when estimating a model), we systematically tested a series of model specifications. This was done first by varying the number of groups and then the order of the trajectory polynomials in order to select the model most optimized for fit and parsimony (64, 66).

Posterior probabilities represent the average probability that the trajectory group each participant was assigned to is the most appropriate group selection and model fit.

This is especially true when multiple mediators are considered in the mediation analysis

**Result section (Prevalence rates of PTSD and trajectory groups by obstetrics and psychosocial variables).** The following paragraphs (sentences) were removed.

The prevalence rates of PTSD symptoms at the 6th, 12th and 18th week of postpartum period were found to be 9.7%, 6.8% and 3.5% respectively.

**Result section (**Predictors of PTSD (PCL-5) trajectory group membership). The following paragraphs (sentences) were removed.

In addition, participants with higher depression and anxiety score were more likely to belong to the PTSD with recovery and chronic PTSD trajectory group. Finally, mothers with stressful life events of relational problems and income instability were also more likely to belong to the PTSD with recovery and chronic PTSD trajectory group.

**Result section (**Mediation analysis of factors associated with PTSD trajectories using the Karlson-Holm-Breen (KHB) method). The following paragraphs (sentences) were removed.

Similarly, the total effect of perceived traumatic childbirth on the PTSD with recovery trajectory group membership, 3.43 (95% CI= 2.10,4.75), could be decomposed into a direct effect, 1.56 (95% CI= 0.33, 2.78), and indirect effect, 1.87 (95% CI= 1.19, 2.55). In relative terms, the indirect effect constituted more than half of (54.58%) of the total effect. Breaking down the indirect effect into its components, it was found that a substantial part of the indirect effect was via WHODAS 2.0 total score (20.14%) anxiety (18.87%) and depression (15.57%) while controlling for other confounders. All mediating effects were statistically significant.

The total effect of fear of childbirth on the PTSD with recovery trajectory group membership, 0.07 (95% CI= 0.04, 0.09), could be also decomposed into a direct effect, 0.04 (95% CI= 0.01, 0.05), and indirect effect, 0.03 (95% CI= 0.02, 0.04). In relative terms, the indirect effect constituted 48.11% of the total effect. Breaking down the indirect effect into its components, it was found that a substantial part of the indirect effect was via anxiety (25.13%) and depression (22.99%). Likewise, the total effect of fear of childbirth on the PTSD vulnerable trajectory group membership, 0.03 (95% CI= 0.02, 0.04), could be decomposed into a direct effect, 0.01 (95% CI= 0.001, 0.02), and indirect effect, 0.02 (95% CI= 0.01, 0.03). In relative terms, the indirect effect constituted more than half of (60.94%) of the total effect. Breaking down the indirect effect into its components, it was found that a substantial part of the indirect effect was via anxiety (36.64%) and depression (24.30%). All mediating effects were statistically significant.

The total and direct effects of WHODAS 2.0 total score on the PTSD with recovery trajectory group membership were 0.24 (95% CI= 0.18, 0.29) and 0.17 (95% CI= 0.12, 0.21), and indirect effect, 0.07 (95% CI= 0.05, 0.10). In relative terms, the indirect effect constituted 30.55% of the total effect. Breaking down the indirect effect into its components, it was found that a substantial part of the indirect effect was via anxiety (19.46%) and depression (11.09%).

The total and direct effects of WHODAS 2.0 total score on the PTSD vulnerable trajectory group membership were 0.12 (95% CI= 0.10, 0.15) and 0.09 (95% CI= 0.07, 0.12), and indirect effect, 0.03 (95% CI= 0.02, 0.05). In relative terms, the indirect effect constituted 27.29% of the total effect. Breaking down the indirect effect into its components, it was found that a substantial part of the indirect effect was via anxiety (19.94%) and depression (7.35%).

The total and direct effects of stressful life event of health risk on the PTSD vulnerable trajectory group membership were 2.10 (95% CI= 1.48, 2.73) and 1.20 (95% CI= 0.58, 1.83), and indirect effect, 0.90 (95% CI= 0.37, 1.44). In relative terms, the indirect effect constituted 42.85% of the total effect. Breaking down the indirect effect into its components, it was found that a substantial part of the indirect effect was via anxiety (30.83%) and depression (12.02%)

**Discussion section.** The following paragraphs (sentences) were removed.

In this study, the prevalence rates of postpartum PTSD symptoms, were found to be 9.7%, 6.8% and 3.5% at the 6^th^, 12^th^ and 18^th^ week of postpartum respectively. This is consistent with a study conducted among perinatal women in Turkey(76). However, 9.7% and 6.8% prevalence of PTSD in our study, is considerably higher than the finding of a systematic review which reported 4% postpartum PTSD(13). The postpartum PTSD symptoms might be due to difficult childbirth experiences triggering new onset of PTSD or exacerbating pre-existing PTSD symptoms. Other possible explanation could be, a difficult birth might retrigger PTSD which was experienced in early life. The prevalence of 3.5% PTSD at the 18^th^ week of postpartum, suggests that PTSD after childbirth might be chronic and indicates that some women with negative birth experiences maintained their negative perception of childbirth and their symptoms until 18^th^ week of postpartum.

**Conclusion section:** The following paragraphs (sentences) were removed.

Prevalence rates of PTSD symptoms were 9.7%, 6.8% and 3.5% at the 6^th^, 12^th^ and 18^th^ week of postpartum respectively.

**I Thank you very much!**
